# Time-critical sepsis interventions and clinical outcomes among children in a tertiary Tanzanian emergency department

**DOI:** 10.1186/s12887-026-07369-2

**Published:** 2026-07-24

**Authors:** Abigail M. Sorensen, Raya Y. Mussa, Erasto M. Sunzula, Victoria M. Mlele, Scott P. Oltman, Cecilia L. Msemwa, Lushona K. Mathias, Kathleen S. Sun, Jazmin M. Baez-Maidana, Juma A. Mfinanga, Victoria T. Chu, Anneka M. Hooft, Namala P. Mkopi, Joel P. Manyahi, Teresa B. Kortz, Hendry R. Sawe

**Affiliations:** 1 Department of Pediatrics, University of California San Francisco, Box 0110, 550 16 St, Floor 4, San Francisco, CA 94143 USA; 2 Muhimbili National Hospital, Dar Es Salaam, Tanzania; 3 Muhimbili University of Health and Allied Sciences, Dar Es Salaam, Tanzania; 4 Department of Epidemiology & Biostatistics, University of California San Francisco, San Francisco, CA USA; 5 University of California San Francisco, Institute for Global Health Sciences, San Francisco, CA USA

**Keywords:** Sepsis, Global health, Early medical intervention, Intensive care units, Pediatric, Resource-limited settings

## Abstract

**Background:**

Pediatric sepsis remains a leading cause of mortality in resource-limited settings. We evaluated the delivery of time-critical sepsis interventions and their association with in-hospital mortality among children in Tanzania.

**Methods:**

We conducted a secondary analysis of a prospective cohort of children (28 days to 14 years) presenting with sepsis to the Emergency Medicine Department at Muhimbili National Hospital, Dar es Salaam, Tanzania (July 2022 to November 2024). Sepsis was defined as fever and at least one World Health Organization warning sign. Local pediatric sepsis guidelines recommend antibiotics and intravenous fluids (IVF) for all patients with sepsis, regardless of the source. Primary exposures were receipt and timing of antibiotics and IVF. The primary outcome was in-hospital mortality. Multivariable logistic regression adjusted for age, nutritional status, referral status, and illness severity.

**Results:**

Among 755 children, 61.2% (*n* = 462) received antibiotics, and 55.6% (*n* = 420) received IVF. Median time to intervention was 2.4 h (IQR 1.6–3.7). 35.5% (*n* = 268) received both interventions, and only 3.7% (*n* = 28) received them within one hour. In-hospital mortality was 19.6% (*n* = 148), with a median time to death of 4.1 days (IQR 1.6–9.5). In a multivariable analysis, time to initiation of both antibiotics and IVF was not significantly associated with in-hospital mortality (adjusted OR 1.29; 95% CI 0.83–2.0; *p* = 0.264). When modeled continuously, a longer time to IVF administration was associated with increased mortality risk (HR 1.05 per hour delay; 95% CI 1.01–1.10).

**Conclusions:**

Receipt of time-critical sepsis interventions (antibiotics and IVF) was delayed and inconsistent, with no observed association with decreased mortality, although residual confounding may remain. This both highlights gaps in early evidence-based care delivery and suggests additional, system-related influences on pediatric sepsis outcomes. Our findings underscore the need for context-specific strategies to strengthen pediatric sepsis management in resource-limited settings.

**Supplementary Information:**

The online version contains supplementary material available at https://doi.org/10.1186/s12887-026-07369-2.

## Background

Sepsis, defined as life-threatening organ dysfunction caused by a dysregulated immune response to infection [1], is a leading cause of pediatric critical illness and death worldwide, accounting for more than one million global child deaths annually [2, 3]. Prompt recognition and treatment of pediatric sepsis has been shown to significantly reduce morbidity and mortality in both high-resource settings (HRS) and resource-limited settings (RLS) [4–6]. Efficient sepsis care is imperative in RLS, as they account for 85% of cases worldwide [2, 7]. This disproportionate burden of sepsis is largely focused in Sub-Saharan Africa (SSA), making it a key region with opportunities for improvement in sepsis care [2, 8]. However, a comprehensive understanding of the local and global sepsis care continuum is necessary to improve outcomes and reduce the burden of disease.

The recently published “Sepsis Chain of Survival” proposes a series of interconnected time-critical interventions as the key components of sepsis treatment to improve sepsis survival across RLS [9]. An extensive HRS-based pediatric study demonstrated that prompt administration of antibiotics and intravenous fluids (IVF) within three hours of sepsis recognition was associated with lower in-hospital mortality and shorter length of hospital stay (LOS) [10]. This is consistent with another study that observed delayed (> 3 h) antibiotic administration was an independent risk factor for in-hospital mortality in septic children [11]. Among time-critical pediatric sepsis interventions, early antibiotic administration has the most compelling evidence for improving patient outcomes; however, the majority of this data originates from HRS and needs further study in RLS [11–14].

While standardized sepsis protocols and timely interventions have been shown to improve outcomes in HRS [10], studies in SSA examining protocols developed in HRS have had mixed results, sometimes associated with worse patient outcomes [15–17]. One potential explanation is that commonly used sepsis tools and interventions are unavailable, delayed, or unreliable in RLS [17–19]. In addition to resource limitations, the pathophysiology of sepsis often differs between RLS and HRS, requiring distinct treatments [2, 7]. This was highlighted in the FEAST trial, which demonstrated an unexpected increase in mortality among septic participants receiving IVF boluses [15]. This underscores the need for continued investigation into the use and effectiveness of current pediatric sepsis management in RLS, ensuring recommendations are consistent with local capabilities, pathophysiology, and resource availability.

In Tanzania, a sub-Saharan nation in East Africa, there are scarce pediatric sepsis data, despite a high burden of disease, with reported in-hospital mortality rates of up to 26.5% [18, 20–23]. Sepsis has been reported as the primary cause of death of critically ill children in Tanzania [24]. These national mortality rates are accompanied by sepsis-related morbidity, providing multiple opportunities for improvement [22]. Critical illness has been well-documented in the Emergency Medicine Departments (EMDs) in Tanzania, and studies have called for greater focus on improving critical sepsis care nationally [25]. The objective of this study was to evaluate treatment, measure the time to delivery of time-critical sepsis interventions (antibiotics and IVF), and determine hospital outcomes of children with sepsis at Muhimbili National Hospital (MNH) in Dar es Salaam, Tanzania.

## Methods

### Study design and population

This is a secondary analysis of a prospective cohort study of children presenting with sepsis conducted at MNH between July 26, 2022, and November 26, 2024. The parent study was designed to characterize the clinical presentation, management, and outcomes of pediatric sepsis at MNH.

MNH, a tertiary care hospital in Dar es Salaam, Tanzania, contains the country’s primary public, 24-h, full-capacity EMD. The EMD has the capacity to treat severely ill children, an existing pediatric sepsis protocol, and the resources to provide time-critical sepsis interventions. The EMD at MNH cares for about 60,000 patients annually, of whom about 25% are pediatric. The EMD is staffed by Emergency Medicine-trained residents, supervising physicians, and nurses. It provides a broad range of emergency care, including plain radiographs, cardiorespiratory monitoring, low-flow oxygen therapy, advanced oxygen support, intubation, mechanical ventilation, and resuscitation medications. The current pediatric sepsis guidelines at MNH recommend that time-critical interventions (antibiotics and IVF) be completed within 1 h (Additional file 1) and that first-line antibiotics be selected according to national recommendations [26]. These interventions have been demonstrated to be time-critical for child survival and considered in both sepsis and septic shock, while other common interventions for pediatric sepsis, such as oxygen or vasoactive support, may be more beneficial in septic shock alone [9–11, 27].

This study included children aged 28 days to 14 years who presented with sepsis, defined as fever (axillary temperature > 38ºC or history of fever), suspected infection, and one or more of the following warning signs: respiratory distress, altered mental status, impaired perfusion, or at-risk for dehydration (Additional file 2) [28]. Sepsis was identified using these predefined clinical criteria; microbiologic confirmation was not required for study inclusion. Other inclusion criteria included having a parent or guardian fluent in English or Swahili. Exclusion criteria included children with acute trauma, burn, cardiac arrest, weight < 4 kg, fever not thought to be due to infection, convulsions with known epilepsy, and those whose guardians were unavailable or declined participation.

### Quantitative variables

#### Data sources and measurements

Participants were recruited using consecutive sampling upon presentation to the EMD and consented by trained research personnel. Once enrolled, study subjects were followed from presentation through hospitalization and until either discharge or death. Participants who transferred to another facility or who absconded were considered to have an outcome of survivor, as they were alive at the time of study site departure. Data were collected by study staff and entered into a secure, electronic REDCap database (version 7.2.2) [29]. This included demographic information, clinical presentation, time of arrival, and timestamps for the initiation of time-critical sepsis interventions (antibiotics and IVF), hospital course, in-hospital mortality, and LOS. The time-to-event data were observed by study staff and manually recorded. Data were de-identified before analysis.

#### Laboratory procedures

All participants underwent blood culture sampling, malaria screening by rapid diagnostic test (RDT) (SD Bioline pan-Pf), and HIV testing by RDT (HIV-1/2 Alere). Positive tests were confirmed by blood smear (malaria) and antibody or PCR testing (HIV). All other testing, treatment, and diagnostic evaluations were at the discretion of the treating clinician, including urine culture, cerebrospinal fluid (CSF) culture, and other diagnostic laboratory tests (e.g., complete blood count, basic metabolic panel, blood gas). To further classify participants’ infectious diagnoses, we used positive urine cultures to diagnose urinary tract infections (UTIs). Pneumonia was diagnosed based on the Integrated Management of Childhood Illness criteria plus a positive finding of focal opacity on chest x-ray (CXR), as reported by local radiologists [30].

Blood cultures were performed in a bacteriology research laboratory at MUHAS using a BD BACTEC FX40 (Becton Dickinson, BD) instrument according to the manufacturer’s instructions. Positive cultures underwent Gram staining and subculturing onto each of the following plates: sheep blood agar (SBA), chocolate agar (CA), and MacConkey agar (MAC). SBA and CA plates were incubated at 35 °C with 5–10% carbon dioxide, and MAC plates were incubated at 37 °C; all plates were incubated for 18–24 h.

*Enterobacterales* and *Staphylococcus aureus *were identified using API20E (BioMérieux, Marcy, I’Etoile, France) and Staphaurex (Remel Europe Ltd, Dartford, United Kingdom), respectively. Antimicrobial susceptibility was determined by the Kirby–Bauer disk diffusion method, following the Clinical and Laboratory Standards Institute guidelines [31]. Contaminants were identified biochemically and genomically. The following organisms were considered contaminants and excluded from analysis: coagulase-negative *Staphylococcus* spp., *Corynebacterium* spp., *Micrococcus* spp., *Bacillus* spp., and *Brevibacterium* spp. The study microbiologist performed quality control and confirmed the results.

#### Sample size

This study was a secondary analysis of a prospective cohort and was not originally designed or powered for the specific hypotheses tested here. The analytic sample included 755 participants, of whom 35.5% (*n* = 268) received both time-critical sepsis interventions. The baseline mortality rate was 19.6% (*n* = 148). Because this was a secondary analysis, effect estimates and 95% confidence intervals were emphasized over formal power estimates.

#### Statistical methods

The primary outcome was in-hospital mortality. The primary exposure was completion of time-critical sepsis interventions, defined as administration of antibiotics and IVF during hospitalization. The secondary exposures were the time-to-event for antibiotic and IVF administration, defined as the time from patient triage to intervention delivery, measured in hours. Our hypothesis was that receipt of time-critical sepsis interventions would decrease in-hospital mortality in children with sepsis presenting to MNH.

We used descriptive statistics to summarize baseline characteristics of the study population, reported categorical variables as frequencies and percentages, and summarized continuous variables as means with standard deviations (SD) or medians with interquartile ranges (IQR), as appropriate. We compared survivors and non-survivors using chi-square tests for categorical variables and t-tests or Wilcoxon rank-sum tests for continuous variables, depending on the data distribution.

We performed bivariate analysis to evaluate the association between the completion of time-critical interventions and in-hospital mortality. We identified *a priori* potential confounders between exposure to interventions and in-hospital mortality based on our prior work [21, 23], literature review [32, 33], and a directed acyclic graph (Additional file 3). All multivariable models, including logistic regression and Cox proportional hazards models, were adjusted for the same a priori selected confounders: age group (< 1 year, 1–2 years, 2–5 years, > 5 years), referral status, nutritional status, and severity of illness (determined by Lambaréné Organ Dysfunction Score [LODS] score, Additional file 4) [34–38]. Nutritional status was classified using age-specific WHO growth standards and z-scores, and anthropometric z-scores outside biologically plausible ranges (± 5 SD) were excluded in accordance with WHO recommendations [39]. From the multivariable models, we calculated individual predicted probabilities of death and used those to calculate the adjusted mortality rate for each group. Adjusted risk differences and 95% CIs were calculated using pairwise contrasts of the predicted probabilities.

We tested the association between time to antibiotics and time to IVF as categorical variables (< 1 h, 1–3 h, > 3 h) [3, 40, 41], defined as the time from triage assessment to initiation of the first antibiotic dose or IVF bolus, and in-hospital mortality using multivariable logistic regression. Time to antibiotic and IVF administration were also modeled as continuous variables (in hours) in Cox proportional hazards models to evaluate for potential dose–response relationships. The proportional hazards assumption was evaluated using Schoenfeld residuals and global tests.

As receipt of antibiotics prior to hospital presentation and referrals from other medical centers are common in this setting and reflective of real-world care pathways, participants who reported receiving antibiotics prior to presentation were not excluded from the primary analysis [42]. In a sensitivity analysis, we evaluated children who did not report receiving antibiotics prior to hospital arrival to assess whether prior treatment influenced associations between time to intervention and mortality. To explore whether referral status modified the association between time-critical intervention receipt and mortality, we conducted referral status-stratified analyses and fitted logistic regression models with interaction terms between intervention receipt and referral status.

For all analyses, statistical significance was defined as a two-sided p-value < 0.05. We reported odds ratios (ORs), HRs, and 95% CIs. All analyses were performed using Stata version 18 (StataCorp, College Station, TX).

#### Bias

To minimize selection bias, consecutive sampling was used to enroll all eligible children presenting to the EMD with sepsis during the study period. Trained research personnel prospectively observed and recorded clinical data and intervention timing using a standardized protocol to reduce information bias. Potential confounding was addressed through multivariable regression, adjusting for key covariates identified *a priori*. To mitigate observer bias, data collectors were not involved in clinical care, and documentation was standardized to minimize subjective interpretation; however, local clinicians were aware of ongoing data collection, which may have led to the Hawthorne effect.

## Results

**Fig. 1:**
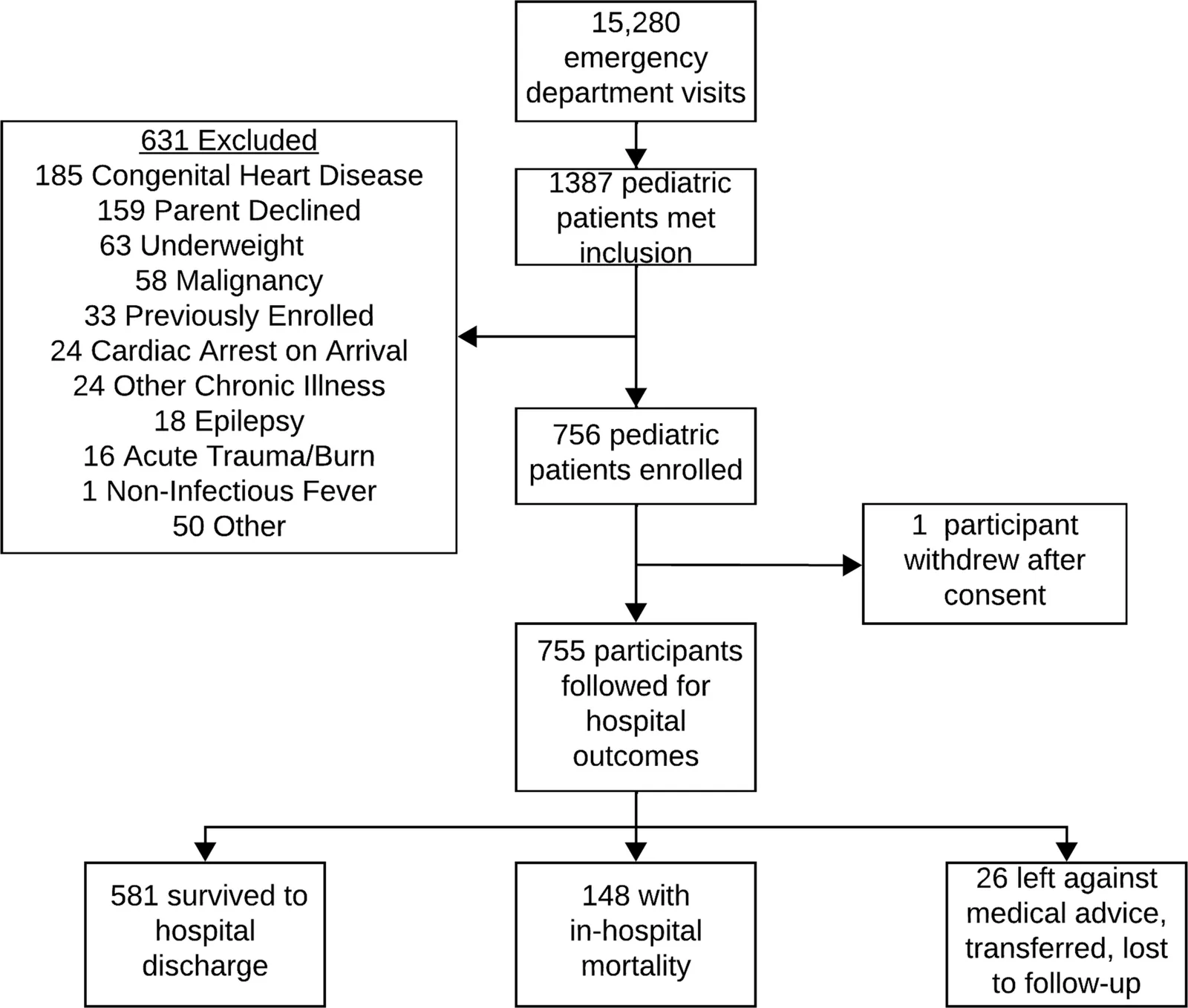
Flowchart depicting participant enrollment and clinical outcomes for Tanzanian children with sepsis

We screened 15,280 children, enrolled 756, and followed 755 for hospital outcomes (Fig. 1). Among participants, the median age was 17 months, 83.0% (*n* = 627) were under 5 years of age, and 56.2% (*n* = 424) were male (Table 1). The prevalence of HIV, malaria, and severe acute malnutrition was 1.6% (*n* = 12), 6.2% (*n* = 47), and 14.4% (*n* = 109), respectively. A total of 36.3% (*n* = 274) of children presented with the lowest severity of illness score, and 27.9% (*n* = 211) had elevated severity of illness (LODS ≥ 2). Among participants, 46.4% (*n* = 350) reported no prior antibiotic use or exposure at the time of hospital presentation. 19.6% (*n* = 148) of children experienced in-hospital mortality, with 7.4% (*n* = 56) experiencing death less than 48 h after hospital presentation. Nutritional status could not be classified for 1.7% (*n* = 13) of participants because all age-appropriate anthropometric indices were unavailable or deemed biologically implausible; these participants were excluded from all logistic models.

**Table 1:** Participant characteristics for children with sepsis in Tanzania and associated in-hospital mortality rates. Missing numbers and percentages are shown only for variables with missing values; there were no missing values for the other variables

Patient characteristics	Total n (%) (*n* = 755)	Survivors n (%) (*n* = 607)	Non-Survivors n (%) (*n* = 148)	*P* value
*Age*				< 0.001
*0–12 months*	272 (36.0)	203 (33.4)	69 (46.6)	
*1–2 years*	181 (24.0)	140 (23.1)	41 (27.7)	
*2–5 years*	174 (23.0)	157 (25.9)	17 (11.5)	
> *5 years*	128 (16.9)	107 (17.6)	21 (14.2)	
*Male Sex*	424 (56.2)	340 (56.0)	84 (56.8)	0.87
*Nutrition Status*				< 0.001
*Moderate Acute Malnutrition*	114 (51.1)	84 (14.0)	30 (20.8)	
*Severe Acute Malnutrition*	109 (14.4)	71 (11.9)	38 (26.4)	
*Missing, n (%)*	13 (1.7)	9 (1.5)	4 (2.7)	
*HIV Positive*	12 (1.6)	5 (0.8)	7 (4.7)	< 0.001
*Fully Vaccinated*	738 (97.7)	593 (97.7)	145 (98.0)	0.78
*Maternal Education Level*				0.005
*No formal school or primary*	276 (36.6)	207 (34.1)	69 (46.6)	
*Secondary/post-secondary*	479 (63.4)	400 (65.9)	79 (53.4)	
*Referred from Outside Location*	411 (54.4)	280 (46.1)	131 (88.5)	< 0.001
Formal Infectious Diagnoses**				
*Bacterial Bloodstream Infection*	40 (5.3)	26 (4.3)	14 (9.5)	0.012
*Urinary Tract Infection*	9 (1.2)	8 (1.6)	1 (0.7)	
*Pneumonia*	135 (17.9)	88 (14.5)	47 (31.8)	< 0.001
*Malaria*	47 (6.2)	36 (5.9)	11 (7.4)	0.44
*Severity of Illness*				< 0.001
*LODS 0*	274 (36.3)	263 (43.3)	11 (7.4)	
*LODS 1*	270 (35.8)	230 (37.9)	40 (27.0)	
*LODS 2*	141 (18.7)	87 (14.3)	54 (36.5)	
*LODS 3*	70 (9.3)	27 (4.4)	43 (29.1)	

*HIV* Human Immunodeficiency Virus, *LODS* Lambaréné Organ Dysfunction Score

^**^Bacterial bloodstream infection was diagnosed via a positive microbial blood culture. Urinary tract infection was diagnosed by a positive urine culture. Pneumonia was diagnosed via the Integrated Management of Childhood Illness guidelines, plus chest x-ray with new focal findings. Malaria was diagnosed by a rapid diagnostic test

Of the patients in our cohort, 5% (*n* = 40) had positive microbial blood culture growth. The most commonly isolated bloodstream pathogen was *Escherichia coli* (*n* = 11), followed closely by *Staphylococcus aureus* (*n* = 10). Among the 36 culture isolates, ceftriaxone would not have been an appropriate antibiotic choice for 31 (86%). This was due to presumed resistance in *Pseudomonas aeruginosa* (*n* = 2), presumed pharmacodynamically suboptimal activity against severe *S. aureus* infections (*n* = 9), and phenotypic resistance identified on antimicrobial susceptibility testing (*n* = 20). We identified resistance to ceftriaxone by pathogen identification and phenotypic antimicrobial sensitivity testing in 45% (*n* = 20) of patients with culture-confirmed pathogens. Of the 20 *Enterobacterales* isolates where antimicrobial susceptibilities were available, resistance to first- and second-line antibiotics for sepsis was common, with resistance rates of 75% to ceftriaxone, 60% to ampicillin, and 65% to gentamicin.

**Fig. 2:**
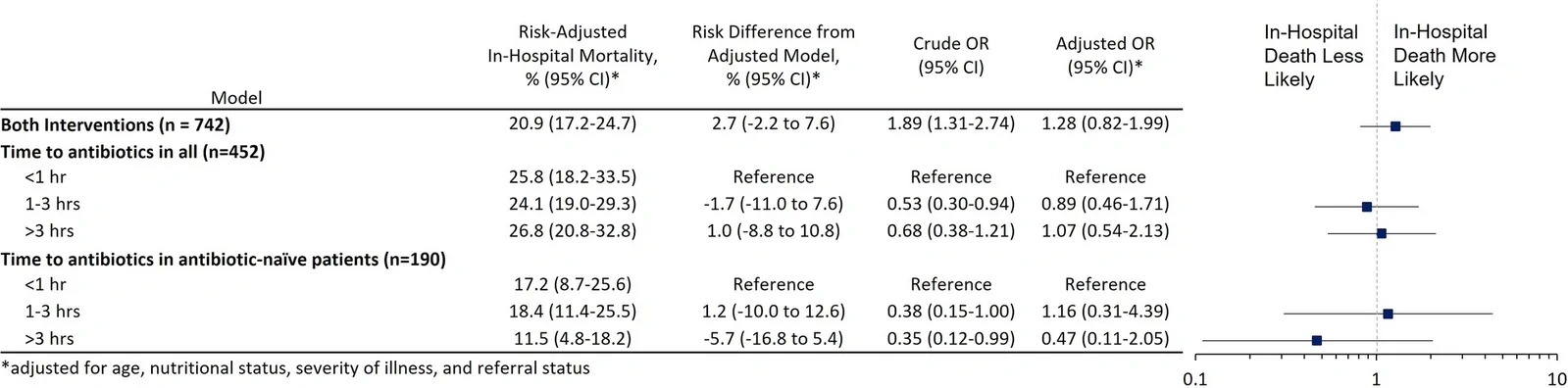
Association between time to antibiotics and in-hospital mortality among Tanzanian children with sepsis. Crude and adjusted odds ratios (ORs) with 95% confidence intervals and model-derived risk-adjusted mortality estimates are shown. Adjusted analyses controlled for age, nutritional status, severity of illness, and referral status

Other infectious etiologies identified in our cohort were UTI (1.2%, *n* = 9) and pneumonia (17.9%, *n* = 135), with mortality more common among children with these diagnoses (Table 1). The most common documented infectious hospital diagnosis and cause of death found in patient charts were acute watery diarrhea (25.7%, *n* = 194) and sepsis (7.9%, *n* = 60), respectively (Additional file 5). Associations between age, infectious diagnoses, intervention timing, and mortality are summarized in Table 1 and the adjusted regression analyses (Fig. 2).

Among all participants, 61.2% (*n* = 462) received antibiotics, and 55.6% (*n* = 420) received IVF during their hospital admission. Both time-critical interventions were provided to 35.5% (*n* = 268) of the cohort during their admission, with a median time to initiation of time-critical sepsis interventions of 2.4 h (IQR 1.6–3.7). The median times to antibiotic administration and IVF were 2.3 h (IQR 1.4–3.7) and 1.8 h (IQR 0.92–3.0), respectively (Table 2). In a multivariable model, completion of the designated time-critical sepsis interventions during hospitalization was not associated with decreased odds of in-hospital mortality (adjusted OR 1.29; 95% CI 0.83–2.0; *p* = 0.264) (Fig. 2).

**Table 2:** Time to event for time-critical interventions in children with sepsis

*Time-Critical Intervention*	*Total n (%) (n* = *755)*	*Median Time to Event (hours)*	*IQR*
*Antibiotics*	462 (61.2)	2.3	1.4–3.7
*IVF*	420 (55.6)	1.8	0.92–3.0

*IQR* Interquartile range, *IVF* Intravenous fluids

In an adjusted multivariable model, we did not observe a significant association between time to antibiotic administration and in-hospital mortality, including among participants who did not receive antibiotics prior to presentation (Fig. 2). Similarly, an adjusted logistic regression analysis for IVF administration demonstrated no significant association between the timing of IVF initiation and in-hospital mortality. Adjusted mortality estimates and adjusted risk differences for each intervention timing group are presented in Fig. 2. When modeled as a continuous variable, time to antibiotic administration was not associated with in-hospital mortality (HR 1.02 per hour delay, 95% CI 0.97–1.07, *p* = 0.55), consistent with the categorical analysis. In contrast, increasing time to IVF administration was associated with increased mortality risk (HR 1.05 per hour delay, 95% CI 1.00–1.10, *p* = 0.029), identifying an association not observed when IVF was modeled categorically.

In an interaction model, the association between time-critical intervention receipt and mortality appeared stronger among self-referred children (OR 3.03, 95% CI 1.00–9.23) than referred children (OR 1.09, 95% CI 0.67–1.75). However, formal interaction testing did not demonstrate statistically significant effect modification by referral status (interaction OR 0.36, 95% CI 0.11–1.20; *p* = 0.096). There was no significant association between antibiotic timing and mortality among either self-referred or referred children. However, in stratified Cox proportional hazards models examining IVF timing as a continuous variable, each additional hour of delay in IVF was associated with increased mortality risk among referred children (hazard ratio [HR] 1.08 per hour, 95% CI 1.00–1.16, *p* = 0.039), but not among self-referred children (HR 0.98, 95% CI 0.89–1.07, *p* = 0.662). Baseline characteristics and outcomes stratified by referral status are presented in Additional file 6.

In adjusted Cox proportional hazards models, increasing illness severity was a strong independent predictor of mortality. Compared with children with LODS 0, those with LODS 3 had a markedly increased hazard of death in both the antibiotic timing model (HR 7.10, 95% CI 2.92–17.28) and the IVF timing model (HR 20.63, 95% CI 6.02–70.63). After adjustment for age, referral status, and nutritional status, time to antibiotic administration was not associated with mortality (HR 1.02 per hour delay, 95% CI 0.97–1.07), whereas increasing time to IVF administration was associated with increased mortality risk (HR 1.05 per hour delay, 95% CI 1.00–1.10) (Additional file 7).

## Discussion

In this cohort of Tanzanian children with sepsis, we observed an in-hospital mortality rate of 19.6%, with fewer than 20% of children receiving the evaluated time-critical sepsis interventions during hospitalization, and only 3.7% receiving them within the recommended timeframe of one hour. Similar mortality rates and delays in care have been reported in pediatric cohorts across SSA and other RLS, underscoring the need for improved adherence to and development of context-adapted guidelines, as well as consideration of non-diagnostic barriers to effective pediatric sepsis care [16, 21, 23, 36].

Barriers to the timely delivery of sepsis interventions have been well documented in RLS and include delayed presentation to care, complex referral systems, cost burden, and inadequate resources [18, 23, 43–46]. Importantly, time to treatment prior to hospital arrival is a major contributor to sepsis-related mortality in these settings. Although we did not directly measure pre-hospital time to treatment, a substantial proportion of our cohort was referred from other facilities, and many participants received care prior to presentation, suggesting that significant delays may have occurred before arrival at the tertiary center. This is supported by published studies describing evaluations and care conducted at peripheral healthcare facilities before patients are referred to tertiary referral centers, resulting in inappropriate and delayed treatment [42, 47]. The Three Delays Model has been applied to patients seeking care in RLS and emphasizes delays in the decision to seek formal healthcare, financial and time barriers to reaching appropriate care, and, finally, the inability to receive proper care due to system or diagnosis delays [47–49]. This delay or history of transfer may have contributed to the observed low rates of time-critical intervention delivery at MNH and influenced clinical decision-making regarding repeat interventions when a prior intervention was reported. This applies to antibiotic delivery, especially given that only 61% of the cohort received antibiotics at MNH. Providers may have withheld antibiotic prescriptions due to participants’ reported medication history. The systemic barriers and upstream delays we discuss, rather than the interventions themselves, may drive poor outcomes and highlight the urgent need to improve both delivery of and access to care [47, 50]. Some participants may report a history of time-critical sepsis intervention receipt, but they may be mistaken, or the reported time interval may be incorrect, leading to further delay in treatment. As this study was not designed to determine the causes of delay in presentation or in the delivery of time-critical interventions, future mixed-methods studies should explore these and other potential reasons for delays in delivering time-critical interventions to septic children in RLS.

Prior studies have demonstrated that delays in time-critical interventions are associated with increased organ dysfunction and mortality in pediatric sepsis [10, 11, 40, 51]. While organ dysfunction was not a primary outcome in our study, severity of illness (LODS) was strongly associated with mortality in our cohort and reflects organ dysfunction, as it evaluates a patient’s respiratory and mental status [52]. Additionally, the high rates of Pediatric Intensive Care Unit admission and advanced critical care interventions, including invasive mechanical ventilation, suggest a substantial burden of organ dysfunction was present in our cohort (Additional file 8).

In addition to demonstrating delays in delivery of time-critical interventions, we found no significant association between time to antibiotic administration and in-hospital mortality. These findings align with prior studies reporting early mortality among participants with sepsis, despite receiving timely antibiotics [14]. This raises important questions about the effectiveness of current antibiotic regimens in this setting, as antibiotics have been shown to be a critical pillar of sepsis management [3]. One potential explanation for the lack of observed association between antibiotic timing and mortality in our study is that many children may have already progressed to advanced disease by the time of presentation, thereby attenuating the impact of early interventions. In contrast to HRS, where early recognition and triage are often feasible, children in RLS frequently experience delays in presentation, referral, and access to advanced treatment.

In contrast to antibiotic timing, we demonstrated an association between time to IVF administration and increased mortality. Notably, in stratified analyses, this association was driven by referred children. This observed association underscores the critical clinical condition of children with sepsis in RLS, likely due to delayed referral and advanced severity of illness at presentation to tertiary referral centers, suggesting every hour of delayed treatment may have detrimental consequences, Frequent upstream delays may result in irreversible organ dysfunction or disease progression, and may render referred children, who have already traversed a longer and more complex care pathway, particularly vulnerable to further delays in resuscitation once they reach tertiary care.

Additionally, differences in antimicrobial selection and resistance patterns between RLS and HRS may further modify the effectiveness of certain time-critical interventions. Previous studies at MNH have demonstrated regional antimicrobial resistance rates of up to 70% among sepsis-causing bacterial pathogens and have suggested that empiric antibiotic selection based on local resistance profiles is a potential context-specific intervention [53–58]. Similar resistance patterns and AMR were observed among participants with BSI in our cohort. The observed lack of association between timely antibiotics and mortality may reflect several factors, including potential mismatch between empiric regimens and local resistance patterns, administration of inappropriate antibiotics prior to referral, and other gaps in sepsis management within the existing protocol.

Our results suggest that the timely delivery of sepsis interventions alone may be insufficient to reduce mortality. Previous studies have raised concerns about the direct application of HRS protocols in RLS, which often assume rapid access to diagnostics, medications, and care [15, 17]. In contrast, sepsis guidelines and locally contextualized protocols have been shown to improve outcomes in other RLS [17, 59]. Current international sepsis protocols, primarily developed in HRS, may not adequately address the realities of RLS, where delays in intervention, resource constraints, and differences in pathogen epidemiology significantly impact outcomes [45, 47, 50, 60].

While this study had many strengths, we acknowledge several potential limitations. First, as a single-center study conducted at a referral hospital, our findings may not be generalizable to other healthcare settings in Tanzania or similar environments. Second, the diagnosis of pediatric sepsis remains challenging in RLS due to the commonly encountered absence of gold-standard diagnostics, which may have led to misclassification or under-identification of cases. Another potential limitation is that the presence of study staff in the EMD may have influenced sepsis management via observer bias. However, we observed poor compliance with time-critical sepsis interventions and believe this bias was unlikely to influence our conclusion. We also may have been limited in our ability to detect small differences in mortality, as this was a secondary analysis. While this is a possibility, our retrospective power analysis indicated that the sample size was adequate to detect a clinically significant difference in mortality. Another potential limitation is the assumption that transferred and absconded patients had a hospital outcome of “survivor”. This was an intentionally conservative assumption that is expected to bias our results towards the null. Next, although we adjusted for severity of illness, residual confounding by indication may remain, as more severely ill children may have preferentially received earlier interventions. Additionally, misclassification bias may have occurred because sepsis was defined by clinical criteria rather than microbiologic confirmation; however, the study definition incorporated fever and WHO warning signs of organ dysfunction to identify children at high risk of severe infection [28, 30]. We did not evaluate the appropriateness of the exposures, including antibiotic choice, dosing, or IVF volume, which may have influenced patient outcomes. Finally, although we attempted to adjust for confounding using illness severity scores and referral status, unmeasured confounders and variability in clinician decision-making may have influenced treatment timing and outcomes.

## Conclusions

This study highlights critical gaps in the delivery of time-critical sepsis interventions in a resource-limited, tertiary hospital setting. Time-critical interventions were inconsistently delivered, frequently delayed, and not associated with improved outcomes among our cohort of children with sepsis. Our findings suggest that timely sepsis interventions alone may not be sufficient and that systemic barriers, including delayed presentation and limited access to care, may also have contributed to these results. Effective pediatric sepsis care requires both prompt treatment at referral centers and a strengthened healthcare system that enables children to reach care without delay. Key priorities for improvement include strengthening adherence to existing local guidelines, identifying and addressing systemic barriers to pediatric sepsis management, and improving the understanding of the local sepsis epidemiology. Future research should prioritize optimizing intervention timing, refining sepsis protocols based on context-specific evidence, and investigating the complex, dynamic healthcare system in RLS. Ultimately, improving the delivery and effectiveness of pediatric sepsis care in RLS will require protocols grounded in the realities of the settings in which they are implemented, rather than adapting HRS models that may not translate effectively to where the burden of pediatric sepsis is greatest.

## Supplementary Information

Additional file 1: Title of data: The pediatric sepsis protocol in the Emergency Department at Muhimbili National Hospital during this study. Description of data: Visual clinical protocol outlining the recognition and initial management of pediatric sepsis in the emergency department, including timing of interventions, fluid resuscitation, antibiotic administration, vasoactive support, and resuscitation goals.

Additional file 2: Title of data: Definitions of Key sepsis terms used to describe and define children with sepsis in Tanzania. Description of data: Operational definitions for key clinical variables and outcomes used in the study, including infection, severe febrile illness, altered mental status, respiratory failure, early mortality, and clinical deterioration, standardized for the Tanzanian pediatric context.

Additional file 3: Title of data: Directed acyclic graph depicting hypothesized relationships among time-critical sepsis care, confounders, and in-hospital mortality. Description of data: This directed acyclic graph illustrates the hypothesized causal relationships between receipt of the time-critical sepsis interventions and in-hospital mortality. The model accounts for potential confounders, including age, severity of illness, referral status, and nutritional status, all of which may influence both the likelihood of receiving the time-critical interventions and mortality risk. The directed acyclic graph was used to guide covariate selection in multivariable regression analyses.

Additional file 4: Title of data: Lambaréné Organ Dysfunction Score. Validated in African children ≤15 years with and without malaria [52]. Description of data: Description of the Lambaréné Organ Dysfunction Score, a simple clinical severity score assigning points for clinical characteristics to quantify organ dysfunction in children.

Additional file 5: Title of data: Most common documented hospital diagnoses and causes of death in children with sepsis in Tanzania. Description of data: Tabulated distribution of hospital diagnoses and causes of death among participants with sepsis, stratified by survival status, based on clinician-documented diagnoses at discharge or death.

Additional file 6: Title of data: Participant characteristics stratified by referral status among Tanzanian children with sepsis. Missing numbers and percentages are shown only for variables with missing values; there were no missing values for the other variables. Description of data: Baseline demographic and clinical characteristics of children with sepsis stratified by referral status. Variables include age, sex, nutritional status, maternal education, infectious diagnoses, illness, and hospital outcomes. Comparisons between self-referred and referred children are presented using descriptive statistics and hypothesis testing.

Additional file 7: Title of data: Association between continuous time to sepsis interventions and in-hospital mortality. Description of data: Cox proportional hazards models adjusted for age group, referral status, nutritional status, and illness severity. Time variables were modeled as continuous variables in hours. Proportional hazards assumptions were met for all models.

Additional file 8: Title of data: Resource utilization in children presenting with sepsis in Tanzania. Description of data: Summary of resource utilization, including respiratory support, vasoactive therapy, imaging, and Pediatric Intensive Care Unit admission, stratified by survival status, with comparisons between survivors and non-survivors.

Additional file 9: Title of data: STROBE reporting guidelines [61]. Description of data: Strengthening the Reporting of Observational Studies in Epidemiology checklist detailing recommended items for transparent reporting of observational studies, including study design, methods, results, and interpretation.

## Data Availability

Study materials, including the template data collection forms, the data dictionary, de-identified data used for all analyses, analytical code, and other study-related materials, will be publicly available at https://borealisdata.ca/dataverse/Pedi_SepsisCoLab. Approval from the lead authors and a signed data access agreement will be required for publications related to these data.

## Abbreviations

HRS: High-resource settings
RLS: Resource-limited settings
SSA: Sub-Saharan Africa
IVF: Intravenous fluids
LOS: Length of stay
MNH: Muhimbili National Hospital
EMD: Emergency Medicine Department
RDT: Rapid diagnostic test
CSF: Cerebrospinal fluid
UTI: Urinary tract infection
CXR: Chest x-ray
MUHAS: Muhimbili University of Health and Allied Sciences
NIMR: National Institute for Medical Research
STROBE: Strengthening the Reporting of Observational Studies in Epidemiology
CI: Confidence interval
SBA: Sheep blood agar
CA: Chocolate agar
MAC: MacConkey agar
IQR: Interquartile range
WHO: World Health Organization
LODS: Lambaréné Organ Dysfunction Score
aOR: Adjusted odds ratio
OR: Odds ratio
HR: Hazard ratio
