# Supplementary material for: Time-critical sepsis interventions and clinical outcomes among children in a tertiary Tanzanian emergency department
Source: BMC Pediatr. 2026 Jul 24;26:1050. doi: 10.1186/s12887-026-07369-2 (PMC13688723; doi:10.1186/s12887-026-07369-2)
Supplement: Supplementary file 1 [file 12887_2026_7369_MOESM2_ESM.pdf]

Additional file 2**:** Definitions of key tepsis terms used to describe and define children with sepsis in Tanzania

**Term**	**Definition**
Altered mental status	‘Verbal’ or less on the Alert-Verbal-Pain-Unresponsive or history of convulsion within the last 24 hours in a patient without known epilepsy.[44]
At-risk for dehydration	The presence of one or more of the following: ≥5 loose stools over the past 24 hours; ≥3 loose stools over past 24 hours and emesis; ≥3 emesis over past 24 hours; decreased urine output per guardian report; or inability to drink or breastfeed.[44,45]
Clinical deterioration	A child who requires intubation, non-invasive positive pressure ventilation, inotropic support, or intensive care unit admission, has a cardiac arrest, or dies within 72 hours of presentation.[46]
Early mortality	Death within 48 hours of presentation.
Increased work of breathing	Presence of retractions, flaring or head bobbing.
Infection	A suspected or proven (positive culture) infection caused by any pathogen **OR** a clinical syndrome associated with a high probability of infection. Evidence for a suspected infection includes: positive findings on clinical exam, imaging, or laboratory tests (e.g., white blood cells in a normally sterile body fluid, perforated viscus, chest radiograph consistent with pneumonia, petechial or purpuric rash, or purpura fulminans).
Respiratory failure	Determined by the presence of **ANY ONE** of the following phrases during a keyword search of physician notes: mechanical ventilation, intubation, ventilator, and bag mask ventilation.
Severe febrile illness	Fever (axillary temperature >38º C or history of fever within 24 hours) with one or more of the following: respiratory distress, altered mental status, impaired perfusion, or at-risk for dehydration.[23]
