# Supplementary material for: Time-critical sepsis interventions and clinical outcomes among children in a tertiary Tanzanian emergency department
Source: BMC Pediatr. 2026 Jul 24;26:1050. doi: 10.1186/s12887-026-07369-2 (PMC13688723; doi:10.1186/s12887-026-07369-2)
Supplement: Supplementary file 2 [file 12887_2026_7369_MOESM3_ESM.pdf]

Additional file 3: Directed acyclic graph depicting hypothesized relationships among time-critical sepsis care, confounders, and in-hospital mortality.

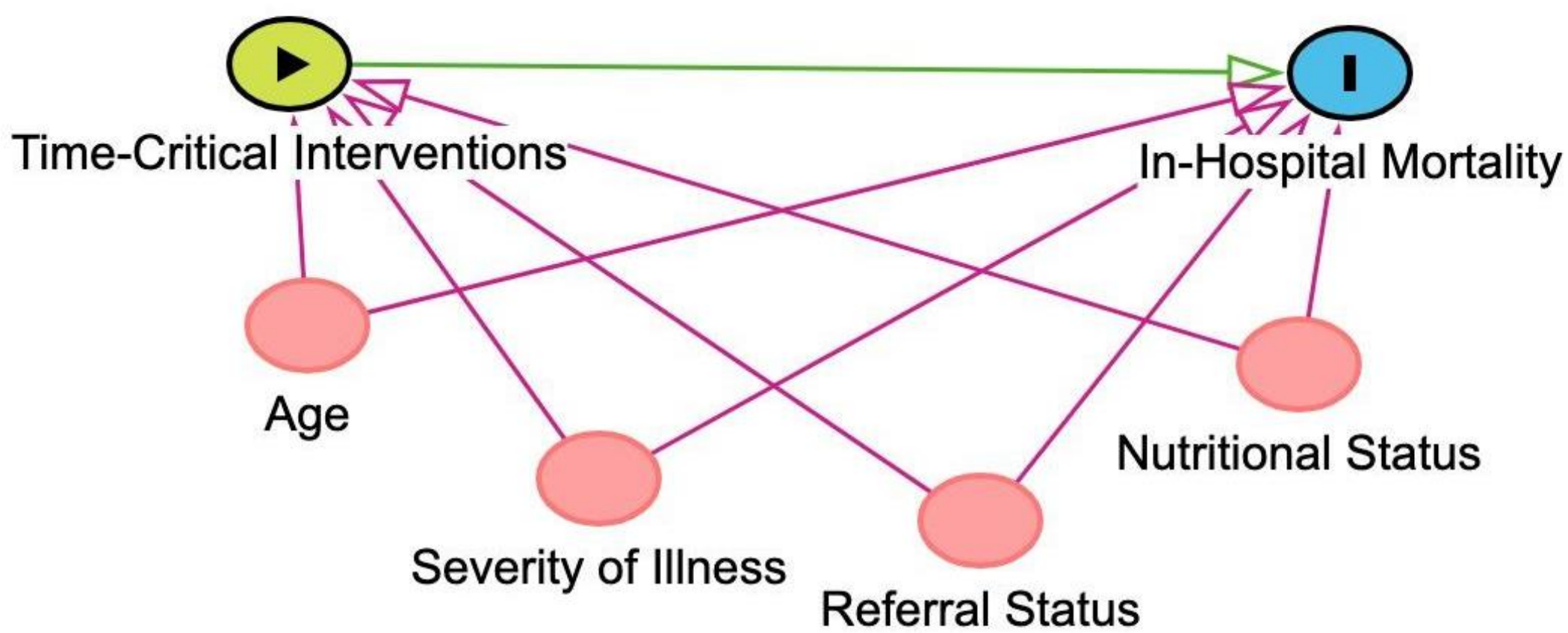

This directed acyclic graph illustrates the hypothesized causal relationships between receipt of the time-critical sepsis interventions and in-hospital mortality. The model accounts for potential confounders, including age, severity of illness, referral status, and nutritional status, all of which may influence both the likelihood of receiving the bundle and mortality risk. The directed acyclic graph was used to guide covariate selection in multivariable regression analyses.
