# Supplementary material for: Time-critical sepsis interventions and clinical outcomes among children in a tertiary Tanzanian emergency department
Source: BMC Pediatr. 2026 Jul 24;26:1050. doi: 10.1186/s12887-026-07369-2 (PMC13688723; doi:10.1186/s12887-026-07369-2)
Supplement: Supplementary file 3 [file 12887_2026_7369_MOESM4_ESM.pdf]

Additional file 4: Lambaréné Organ Dysfunction Score. Validated in African children ≤15 years with and without malaria.[61]

**Lambaréné Organ Dysfunction Score (LODS)**	
The score assigns one point for each item:	● Prostration – defined as ≥1 of the following, depending on age: inability to breastfeed, sit, stand, or walk unsupported<br>● Coma – Blantyre Coma Score ≤2<br>● Deep breathing
