# Supplementary material for: Time-critical sepsis interventions and clinical outcomes among children in a tertiary Tanzanian emergency department
Source: BMC Pediatr. 2026 Jul 24;26:1050. doi: 10.1186/s12887-026-07369-2 (PMC13688723; doi:10.1186/s12887-026-07369-2)
Supplement: Supplementary file 4 [file 12887_2026_7369_MOESM5_ESM.pdf]

Additional file 5: Most common documented hospital diagnoses and causes of death in children with sepsis in Tanzania.

Hospital Diagnosis*	***Survivors n, (%) (n = 607)***	***Non-Survivors n, (%) (n = 148)***
Acute watery diarrhea	186 (31)	8 (5)
Bronchiolitis	13 (2)	0 (0)
Fever of Unknown Origin	4 (1)	0 (0)
HIV/AIDS	3 (1)	11 (7)
Hepatitis	2 (0)	0 (0)
Malaria	28 (5)	6 (4)
Measles	11 (2)	0 (0)
Meningitis/Encephalitis	38 (6)	12 (8)
Non-infectious diagnosis	163 (27)	30 (20)
Other	5 (1)	2 (1)
Pneumonia	68 (11)	15 (10)
Respiratory infection	3 (1)	0 (0)
SSTI	3 (1)	0 (0)
Sepsis	33 (6)	60 (41)
Tuberculosis	3 (1)	4 (3)
Upper Respiratory Infection	28 (5)	0 (0)
Urinary Tract Infection	14 (2)	0 (0)

HIV: Human Immunodeficiency Virus; AIDS: Acquired Immune Deficiency Syndrome; SSTI: Skin and Soft Tissue Infection

**These diagnoses were provided by the clinical team at the time of hospital discharge or patient death and do not reflect the clinical laboratory findings or our study's definition of sepsis.*
