# Supplementary material for: Time-critical sepsis interventions and clinical outcomes among children in a tertiary Tanzanian emergency department
Source: BMC Pediatr. 2026 Jul 24;26:1050. doi: 10.1186/s12887-026-07369-2 (PMC13688723; doi:10.1186/s12887-026-07369-2)
Supplement: Supplementary file 5 [file 12887_2026_7369_MOESM6_ESM.pdf]

Additional file 6: Participant characteristics stratified by referral status among Tanzanian children with sepsis. Missing numbers and percentages are shown only for variables with missing values; there were no missing values for the other variables.

Patient Characteristics	***Total n (%) (n=755)***	***Self-Referred n (%) (n=343)***	***Referred n (%) (n=411)***	***P value***
Age				<0.001
0-12 months	272 (36.0)	98 (28.6)	174 (42.3)	
1-2 years	181 (24.0)	75 (21.9)	105 (25.5)	
2-5 years	174 (23.0)	102 (29.7)	72 (17.5)	
> 5 years	128 (16.9)	68 (19.8)	60 (14.6)	
Male Sex	424 (56.2)	198 (57.7)	225 (54.7)	0.41
Nutrition Status				0.12
Moderate Acute Malnutrition	114 (15.1)	43 (12.8)	71 (17.5)	
Severe Acute Malnutrition	108 (14.3)	41 (12.2)	67 (16.5)	
Missing, n (%)	13 (1.7)	7 (2.0)	6 (1.5)	
HIV Positive	12 (1.6)	2 (0.6)	10 (2.4)	0.043
Fully Vaccinated	738 (97.7)	339 (98.8)	398 (96.8)	0.14
Maternal Education Level				<0.001
No formal or primary education	276 (36.6)	85 (24.8)	191 (46.5)	
Secondary or post-secondary education	479 (63.4)	258 (75.2)	220 (53.5)	
Formal Infectious Diagnoses*				
Bacterial Bloodstream Infection	40 (5.3)	21 (6.1)	19 (4.6)	0.36
Urinary Tract Infection	9 (1.2)	4 (1.2)	5 (1.2)	
Pneumonia	135 (17.9)	21 (6.1)	114 (27.7)	<0.001
Malaria	47 (6.2)	15 (4.4)	32 (7.8)	0.065
Severity of Illness				<0.001
LODS 0	274 (36.3)	189 (55.1)	85 (20.7)	
LODS 1	270 (35.8)	130 (37.9)	140 (34.1)	
LODS 2	141 (18.7)	15 (4.4)	125 (30.4)	
LODS 3	70 (9.3)	9 (2.6)	61 (14.8)	

HIV: Human Immunodeficiency Virus; LODS: Lambaréné Organ Dysfunction Score

**Bacterial bloodstream infection was diagnosed via a positive microbial blood culture. Urinary tract infection was diagnosed by a positive urine culture. Pneumonia was diagnosed via the Integrated Management of Childhood Illness guidelines, plus chest x-ray with new focal findings. Malaria was diagnosed by a rapid diagnostic test.*
