# Supplementary material for: Time-critical sepsis interventions and clinical outcomes among children in a tertiary Tanzanian emergency department
Source: BMC Pediatr. 2026 Jul 24;26:1050. doi: 10.1186/s12887-026-07369-2 (PMC13688723; doi:10.1186/s12887-026-07369-2)
Supplement: Supplementary file 6 [file 12887_2026_7369_MOESM7_ESM.pdf]

Additional file 7: Association between continuous time to sepsis interventions and in-hospital mortality

Variable	***Adjusted HR***	***95% CI***	***P value***
Time to Antibiotics *(per hour delay)*	1.02	0.97-1.07	0.550
Time to Intravenous Fluids *(per hour delay)*	1.05	1.00-1.10	0.029

HR: hazard ratio; CI: confidence interval

Cox proportional hazards models adjusted for age group, referral status, nutritional status, and illness severity. Time variables were modeled as continuous variables in hours.
