# Supplementary material for: Time-critical sepsis interventions and clinical outcomes among children in a tertiary Tanzanian emergency department
Source: BMC Pediatr. 2026 Jul 24;26:1050. doi: 10.1186/s12887-026-07369-2 (PMC13688723; doi:10.1186/s12887-026-07369-2)
Supplement: Supplementary file 7 [file 12887_2026_7369_MOESM8_ESM.pdf]

Additional file 8: Resource utilization in pediatric patients presenting with sepsis in Tanzania.

Resource	***Total n, (%)***	***Survivors n, (%) (n = 607)***	***Non-Survivors n, (%) (n = 148)***	***P value***
Supplemental oxygen	220 (29)	117 (19)	103 (70)	<0.001
Any non-invasive mechanical ventilation				0.16
CPAP	5 (1)	3 (0.5)	2 (1.4)	
Bubble CPAP	6 (1)	1 (0.2)	5 (3.4)	
BiPAP	1 (0)	1 (0.2)	0 (0)	
Bag valve mask ventilation	2 (0.3)	0 (0)	2 (1.4)	
Invasive mechanical ventilation	114 (15)	39 (6.0)	75 (51)	<0.001
PICU admission	190 (25)	88 (15)	102 (69)	<0.001
Vasoactive support	63 (8)	15 (3)	48 (32)	<0.001
Imaging studies				
X-ray	269 (36)	193 (32)	76 (51)	<0.001
CT	34 (5)	24 (4)	10 (7)	0.14
MRI	16 (2)	13 (2)	3 (2)	0.93
Ultrasound	83 (11)	73 (12)	10 (7)	0.07

CPAP: continuous positive airway pressure, BiPAP: bilevel positive airway pressure, PICU: Pediatric Intensive Care Unit, CT: computed tomography, MRI: magnetic resonance imaging
